# Circular RNA circMBOAT2 promotes prostate cancer progression via a miR-1271-5p/mTOR axis

**DOI:** 10.18632/aging.103432

**Published:** 2020-07-09

**Authors:** Juanyi Shi, Cheng Liu, Changhao Chen, Kaixuan Guo, Zhuang Tang, Yuming Luo, Luping Chen, Yinjie Su, Kewei Xu

**Affiliations:** 1 Department of Urology, Sun Yat-sen Memorial Hospital, Sun Yat-sen University, Guangzhou, Guangdong, P. R. China; 2 Guangdong Provincial Key Laboratory of Malignant Tumor Epigenetics and Gene Regulation, Sun Yat-sen Memorial Hospital, Sun Yat-sen University, Guangzhou, Guangdong, P. R. China; 3 Department of Pancreatobiliary Surgery, Sun Yat-sen Memorial Hospital, Sun Yat-sen University, Guangzhou, Guangdong, P. R. China; 4 Department of Pediatric Surgery, Sun Yat-sen Memorial Hospital, Sun Yat-sen University, Guangzhou, Guangdong, P. R. China

**Keywords:** circMBOAT2, mTOR, PI3K/Akt, prostate cancer, metastasis

## Abstract

Patients with advanced prostate cancer (PCa) have poor prognosis. Circular RNAs (circRNAs) regulate biological processes in a variety of cancers, but the precise roles of circRNAs in PCa are poorly understood. Herein, we identified a novel circRNA, termed circMBOAT2 (has_circ_0007334), which was significantly overexpressed in PCa tissues and cell lines. Overexpression of circMBOAT2 was associated with high Gleason score, advanced pathological T stage, and poor prognosis. Overexpression of circMBOAT2 promoted proliferation, migration, and invasion of PCa cells *in vitro*, and enhanced tumorigenesis and metastasis *in vivo*. Mechanistically, circMBOAT2 overexpression upregulated the expression of mTOR by acting as a decoy for miR-1271-5p, resulting in the activation of the PI3K/Akt pathway, ultimately promoting the progression of PCa. Importantly, application of an inhibitor of mTOR significantly antagonized circMBOAT2-mediated PCa tumorigenesis *in vivo*. circMBOAT2 promotes proliferation and metastasis of PCa through miR-1271-5p/mTOR axis-mediated activation of the PI3K/Akt pathway. In summary, our findings uncover a molecular mechanism in the progression of PCa and indicate that circMBOAT2 may be a useful prognostic biomarker and therapeutic target in PCa.

## INTRODUCTION

Prostate cancer (PCa) is the second most common cancer and the most common urinary malignancy worldwide, with approximately 1.3 million new cases and 360,000 associated deaths worldwide in 2018 [1]. Although there are many strategies for the treatment of early stage PCa, almost 30% of the cases eventually metastasize, which drastically shortens the overall survival of PCa patients [2–4]. Therefore, a better understanding of the precise molecular mechanisms underlying PCa development and metastasis is necessary to improve survival for patients with metastatic PCa.

Circular RNAs (circRNAs) are a novel class of noncoding RNAs that are formed by covalent closed-loop RNA structures lacking 5’ caps and 3’ polyadenylation tails [5]. circRNAs are evolutionarily conserved, stable, abundant, and tissue-specific, and their regulatory effects have been shown to participate in the initiation and progression of cancer [6, 7]. circRNAs regulate gene expression by acting as miRNA sponges or interacting with RNA-binding proteins, and some circRNAs encode functional proteins [8–10]. A recent study has revealed widespread expression and significant involvement of circRNAs in PCa [11]. However, the biological functions and mechanisms of circRNAs in PCa have not been explored comprehensively.

Activation of the phosphatidylinositol 3-kinase (PI3K)/Akt pathway promotes malignant transformation, proliferation, and metastasis in cancer, and the PI3K/Akt pathway is frequently activated in PCa, [12–14]. Indeed, alterations the PI3K/Akt pathway occur in 42% of localized prostate tumors and in 100% of metastatic prostate tumors [15, 16]. The mammalian target of rapamycin (mTOR) kinase, one of the phosphatidylinositol kinase-related kinases, plays an essential role in PI3K/Akt pathway activation through assembling mTOR-complex 1 (mTORC1) and mTOR-complex 2 (mTORC2) in PCa [17–20]. Therefore, exploring the molecular mechanisms underlying mTOR upregulation and tumor progression may inform strategies to use anti-mTOR treatments in PCa.

In the present study, we discovered that the circRNA circMBOAT2 (has_circ_0007334) was overexpressed in human PCa tissues. We found that overexpression of circMBOAT2 was associated with poor prognosis among patients with PCa. Mechanistically, circMBOAT2 promoted PCa proliferation and metastasis by sponging miR-1271-5p, resulting in upregulation of mTOR expression and further activating the PI3K/Akt signaling pathway. Our findings revealed that circMBOAT2 is a novel oncogenic circRNA that is a potential prognostic biomarker and therapeutic target in PCa.

## RESULTS

### Identification and characterization of circMBOAT2 in PCa cells

**Figure 1:**
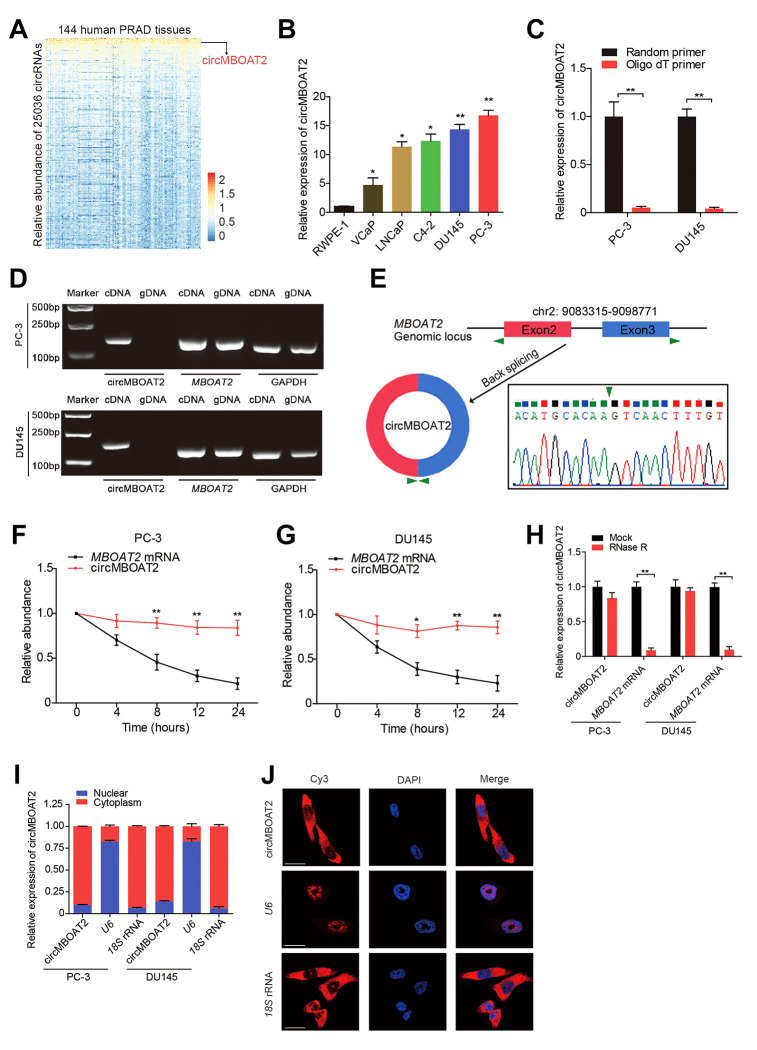
**Identification and characteristics of circMBOAT2 in PCa cells.** (**A**) A heatmap showing circMBOAT2 ranks among the top 1% most enriched circRNAs in 144 cases of prostate cancer tissues (GSE113120). (**B**) qRT-PCR analysis of circMBOAT2 expression in PCa cell lines. (**C**) qRT-PCR analysis of circMBOAT2 abundance using oligo-dT primers and random primers in cDNA synthesis. (**D**) Gel electrophoresis for PCR products of circMBOAT2 and linear *MBOAT2* derived from cDNA and gDNA. (**E**) The formation of circMBOT2, derived from exons 2 and 3 of the *MBOAT2* gene. The back-splice junction of circMBOAT2 was confirmed by Sanger sequencing. (**F**, **G**) qRT-PCR analysis of the abundance of circMBOAT2 and *MBOAT2* in PC-3 and DU145 cells treated with actinomycin D at the indicated times. (**H**) qRT-PCR analysis of the abundance of circMBOAT2 and *MBOAT2* in PC-3 and DU145 cells treated with or without RNase R. (**I**) qRT-PCR analysis of circMBOAT2 abundance in the nuclear and cytoplasmic fractions of PC-3 and DU145 cells. (**J**) Fluorescence in situ hybridization (FISH) for cellular distribution of circMBOAT2 in PC-3 cells. Scale bar: 20 μm. Data are displayed as mean ± SD. *p < 0.05; **p < 0.01.

To identify the critical circRNAs involved in PCa progression, we analyzed published RNA-seq data (GSE113120) from human PCa tissue and found that circMBOAT2 was highly enriched in PCa (Figure 1A). Assessment of circMBOAT2 expression in prostate cancer and normal prostate cell lines showed that circMBOAT2 was significantly overexpressed in PCa cell lines (VCaP, LNCaP, C4-2B, DU145 and PC-3) compared to the normal prostate epithelial cell line RWPE-1 (Figure 1B). To verify the circular structure of circMBOAT2, we designed divergent primers to amplify the circMBOAT2 form and convergent primers to amplify another exon of the linear *MBOAT2* mRNA. PCR and agarose gel electrophoresis showed that the circular form was detected in cDNA, while no amplification product was obtained from gDNA when using divergent primers (Figure 1D). Sanger sequencing of the PCR product determined the head to tail splicing of circMBOAT2, which was consistent with the results obtained from circBase, and indicated that circMBOAT2 was derived from exon 2 and exon 3 of the *MBOAT2* gene (Figure 1E), supporting the closed circular structure of circMBOAT2. Moreover, reverse transcription experiments showed that circMBOAT2 was rarely detected when oligo-dT primers were used, indicating the deletion of the 3’ poly(A) tail in circMBOAT2 (Figure 1C). Meanwhile, circMBOAT2 was resistant to digestion by RNase R, a 3′to 5′ exoribonuclease, whereas the linear *MBOAT2* mRNA showed sensitivity to RNase R treatment (Figure 1H), further demonstrating that circMBOAT2 exists as a circular RNA in PCa. Furthermore, an actinomycin D assay was preformed to verify the transcript half-life of circMBOAT2, which exceeded 24 h (Figure 1F, 1G), suggesting that the circular form is more stable than the linear mRNA counterpart. Subcellular fractionation assay and fluorescence in situ hybridization (FISH) indicated that circMBOAT2 is predominantly distributed in the cytoplasm (Figure 1I, 1J). These results demonstrate that circMBOAT2 is overexpressed in PCa cell lines and mainly localized in the cytoplasm as a highly stable circRNA.

### circMBOAT2 promotes PCa cell proliferation, migration, and invasion *in vitro*

**Figure 2:**
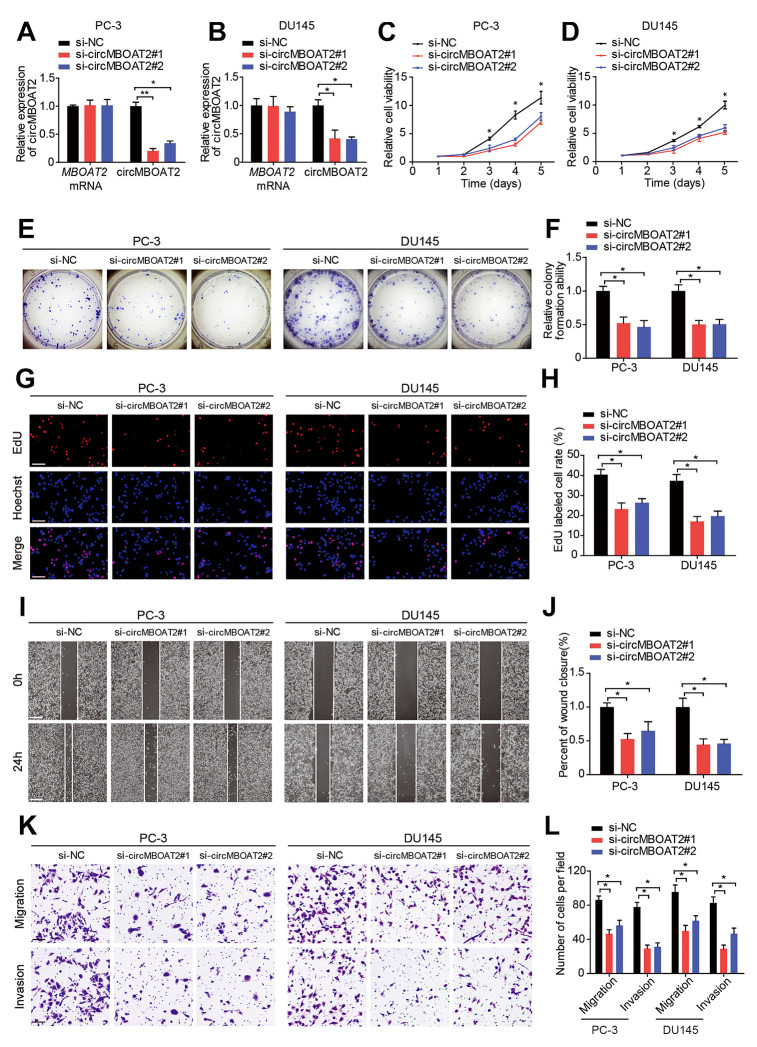
**circMBOAT2 promotes cell proliferation, migration, and invasion *in vitro.*** (**A**, **B**) qRT-PCR analysis of circMBOAT2 and *MBOAT2* expression in PC-3 and DU145 cells treated with circMBOAT2 siRNAs. (**C**, **D**) CCK-8 assay determined the cell viability in PC-3 and DU145 cells treated with circMBOAT2 siRNAs. (**E**, **F**) Representative images and quantifications of colony formation assays in PC-3 and DU145 cells treated with circMBOAT2 siRNAs. (**G**, **H**) Representative images and quantifications of EdU assays in PC-3 and DU145 cells treated with circMBOAT2 siRNAs. Scale bars: 100 μm. (**I**, **J**) Representative images and quantifications of wound healing assays in PC-3 and DU145 cells treated with circMBOAT2 siRNAs. Scale bars: 200 μm. (**K**, **L**) Representative images and quantification of transwell assays in PC-3 and DU145 cells treated with circMBOAT2 siRNAs. Scale bars: 100 μm. Data are displayed as mean ± SD. *p < 0.05; **p < 0.01.

We next explored the functions of circMBOAT2 in PCa cells. The expression of circMBOAT2 was successfully downregulated or overexpressed in PC-3 and DU145 cells through siRNA (Supplementary Table 3) or expression plasmid transfection, respectively (Figure 2A, 2B, Supplementary Figure 1A, 1B). Transfection of the circMBOAT2 siRNA or expression vectors did not alter the linear MBOAT2 mRNA expression. CCK-8, colony formation, and EdU assays indicated that knockdown of circMBOAT2 inhibited cell proliferation in both PC-3 and DU145 cells (Figure 2C–2H). Overexpression of circMBOAT2 promoted cell proliferation (Supplementary Figure 1C–1H). Furthermore, wound healing and transwell assays demonstrated that the migratory and invasive capability of PCa cells were significantly attenuated with circMBOAT2 depletion (Figure 2I–2L), and enhanced after circMBOAT2 overexpression (Supplementary Figure 1I–1L). These data demonstrate that circMBOAT2 promotes proliferation, migration, and invasion of PCa cells *in vitro*.

### circMBOAT2 enhances the tumorigenesis and metastasis of xenograft tumors *in vivo*

**Figure 3:**
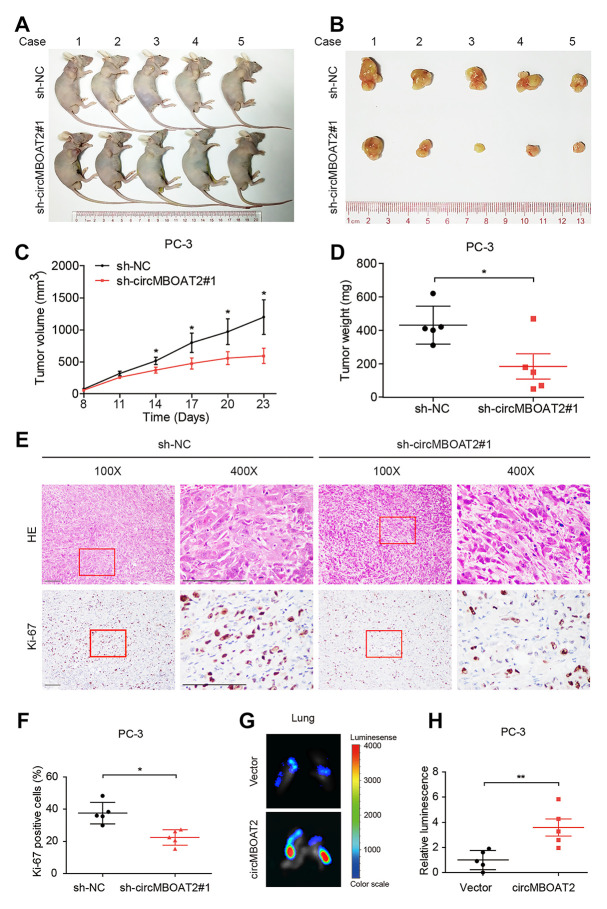
**circMBOAT2 promotes tumorigenesis and metastasis *in vivo.*** (**A**, **B**) Images of the xenograft subcutaneous models of PC-3 cells stably transfected with sh-circMBOAT2#1 or sh-NC. (**C**, **D**) Tumor volumes and weights of subcutaneous xenograft tumors. (**E**, **F**) Representative images of IHC staining and analysis of Ki-67 expression. Scale bars: 100 μm. (**G**, **H**) Representative bioluminescence images and analysis of excised lungs from xenograft metastatic models. Data are displayed as mean ± SD. *p < 0.05; **p < 0.01.

To explore the effects of circMBOAT2 *in vivo*, PC-3 cells stably expressing firefly luciferase were established. Luciferase-labeled PC-3 cells were transfected with sh-circMBOAT2 or sh-NC and were subcutaneously injected into BALB/c nude mice (Figure 3A, 3B). The volume and weight of tumors were significantly decreased in the sh-circMBOAT2 group compared with the sh-NC group (Figure 3C, 3D). Immunohistochemistry revealed that circMBOAT2 silencing reduced the number of Ki-67-positive cells (Figure 3E, 3F), suggesting that suppression of circMBOAT2 impaired proliferation of PCa cells *in vivo*.

To investigate the effects of circMBOAT2 on PCa metastasis *in vivo*, an intravenous injection metastatic model was employed. Luciferase-labeled PC-3 cells were transfected with circMBOAT2 or vector and were injected into the tail vein of nude mice. We found that the luminescence from labeled cells in the lungs was pervasive in the circMBOAT2 overexpressing group, but weaker or undetectable in the vector control group (Figure 3G, 3H). These results indicate that circMBOAT2 may promote metastasis of PCa cells *in vivo*.

### circMBOAT2 serves as a miRNA sponge for miR-1271-5p in PCa cells

**Figure 4:**
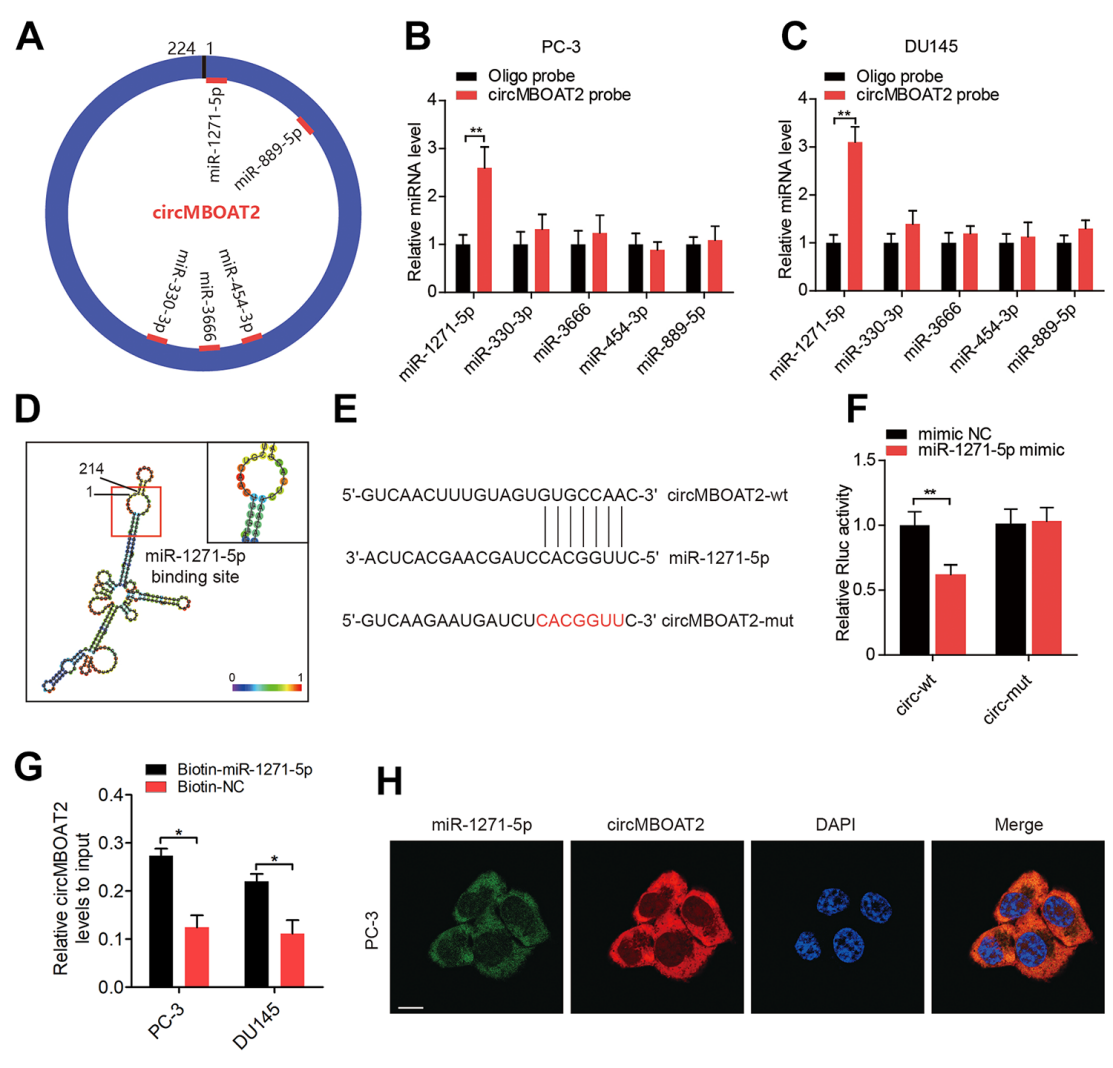
**circMBOAT2 functions as a sponge for miR-1271-5p.** (**A**) A schematic diagram showing potential target miRNAs of circMBOAT2. (**B**, **C**) RNA pull-down assay revealed that circMBOAT2 interacts with miR-1271-5p in PC-3 and DU145 cells (**D**) RNAalifold predicted the binding site of miR-1271-5p on circMBOAT2. (**E**) Wild-type (wt) and mutated (mut) sequences of the predicted binding site between circMBOAT2 and miR-1271-5p. (**F**) Dual-luciferase reporter assays revealing binding properties of circMBOAT2 and miR-1271-5p. *Renilla* luciferase (Rluc) activity was normalized to firefly luciferase activity. (**G**). qRT-PCR analysis of the abundance of circMBOAT2 captured by biotin-labeled microRNA probes. (**H**) FISH assay showed the colocalization of circMBOAT2 and miR-1271-5p. Data are displayed as mean ± SD. *p < 0.05; **p < 0.01.

circRNAs can act as RNA sponges to interact with miRNAs in the cytoplasm [5]. As we had validated the cytoplasmic localization of circMBOAT2 in PCa cells, we determined whether circMBOAT2 interacted with miRNAs as a miRNA sponge in PCa. Predictions from three independent miRNA target databases (MiRanda, TargetScan, and RNAhybird) were compared, and five miRNAs (miR-1271-5p, miR-330-3p, miR-3666, miR-454-3p and miR-889-5p) were commonly predicted as potential binding partners of circMBOAT2 (Figure 4A). RNA pull-down assay with biotin-labeled circMBOAT2 probe and oligo probes showed that circMBOAT2 exhibited an adsorption affinity for miR-1271-5p in both PC-3 and DU145 cells (Figure 4B, 4C). Sequence analysis by RNAalifold [21] indicated that the miR-1271-5p binding site was located in the 1-7 nt region of circMBOAT2 (Figure 4D). To further validate the binding sequences of miR-1271-5p on circMBOAT2, we co-transfected miR-1271-5p mimics or non-targeting mimic (mimic NC) and a luciferase reporter containing the sequences of wild type or mutated circMBOAT2 into HEK 293T cells (Figure 4E). Compared with the mimic NC, miR-1271-5p significantly reduced the Rluc activity of the circMBOAT2-wt reporter, while no significant change was obtained when the binding site of miR-1271-5p was mutated, indicating these sequences represent regions of interaction between circMBOAT2 and miR-1271-5p (Figure 4F). Moreover, RNA capture assays revealed enrichment of circMBOAT2 with biotin-labeled miR-1271-5p, further confirming that circMBOAT2 interacted with miR-1271-5p (Figure 4G). Furthermore, FISH confirmed that circMBOAT2 and miR-1271-5p co-localized in the cytoplasm (Figure 4H). Together, these results suggest that circMBOAT2 functions as a miRNA sponge through direct targeting of miR-1271-5p.

### miR-1271-5p acts as a tumor suppressor and targets mTOR in PCa cells

**Figure 5:**
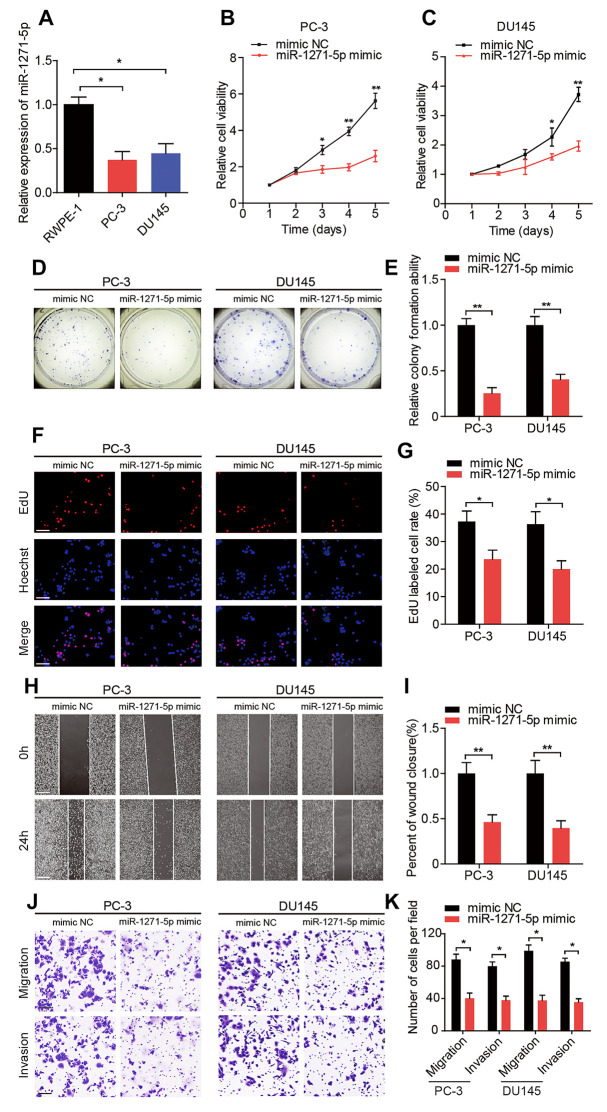
**miR-1271-5p functions as a tumor suppressor *in vitro.*** (**A**) qRT-PCR analysis of miR-1271-5p expression in RWPE-1, PC-3, and DU145 cells. (**B**, **C**) CCK-8 assay determined the cell viability in PC-3 and DU145 cells treated with miR-1271-5p mimic. (**D**, **E**) Representative images and quantification of colony formation assays in PC-3 and DU145 cells treated with miR-1271-5p mimic. (**F**, **G**) Representative images and quantification of EdU assays in PC-3 and DU145 cells treated with miR-1271-5p mimic. Scale bars: 100 μm. (**H**, **I**) Representative images and quantification of wound healing assays in PC-3 and DU145 cells treated with miR-1271-5p mimic. Scale bars: 200 μm. (**J**, **K**) Representative images and quantifications of transwell assays in PC-3 and DU145 cells treated with miR-1271-5p mimic. Scale bars: 100 μm. Data are displayed as mean ± SD. *p < 0.05; **p < 0.01.

Given that circMBOAT2 acted as a sponge for miR-1271-5p, we next explored the function and downstream targets of miR-1271-5p in PCa cells. We found that miR-1271-5p was downregulated in human PCa cell lines compared with the RWPE-1 cells (Figure 5A). Moreover, miR-1271-5p overexpression significantly repressed the proliferation, migration, and invasion of PCa cells compared with the mimic NC (Figure 5B–5K). In contrast, the proliferation, migration, and invasion of PCa cells were enhanced when PCa cells were treated with a miR-1271-5p inhibitor (Supplementary Figure 2A–2J). In addition, the expression of miR-1271-5p in PCa tissues and matched normal adjacent tissues was detected by qRT-PCR. The results showed that the expression of miR-1271-5p was significantly downregulated in PCa tissues compared with matched normal adjacent tissues and lower miR-1271-5p expression levels were significantly correlated with shorter disease-free survival (DFS) (Supplementary Figure 3A, 3B). Moreover, we found a negative correlation between circMBOAT2 and miR-1271-5p expression, which suggesting that circMBOAT2 acts as a sponge for miR-1271-5p in PCa tissues (Supplementary Figure 3C).

**Figure 6:**
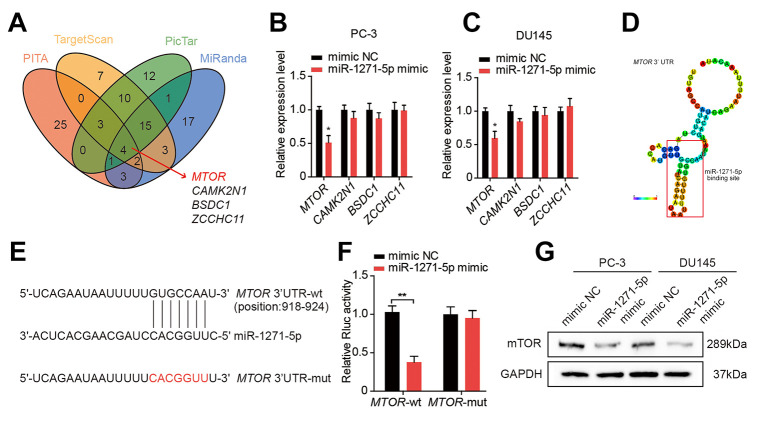
**miR-1271-5p targets mTOR in PCa cells.** (**A**) Target genes of miR-1271-5p were predicted by Starbase 2.0. (**B**, **C**) qRT-PCR analysis of the expression of target genes in PC-3 and DU145 cells treated with the miR-1271-5p mimic. (**D**) RegRNA 2.0 predicted the binding site of miR-1271-5p on circMBOAT2. (**E**) Wild type (wt) and mutated (mut) sequences of the predicted binding site between the *MTOR* 3’UTR and miR-1271-5p. (**F**) Dual-luciferase reporter assays showing the binding properties of *MTOR* and miR-1271-5p. *Renilla* luciferase (Rluc) activity was normalized to firefly luciferase activity. (**G**) Western blot analysis of mTOR expression in PC-3 and DU145 cells treated with miR-1271-5p. Data are displayed as mean ± SD. *p < 0.05; **p < 0.01.

We applied bioinformatics analysis to find putative regulatory targets of miR-1271-5p, and we identified an intersection set containing four genes (Figure 6A). qRT-PCR showed that miR-1271-5p overexpression decreased the expression of *MTOR* (Figure 6B, 6C), which contained specific sequences on its 3'UTR complementary to the seed sequence of miR-1271-5p, based on the predictive results from RegRNA2.0 [22] (Figure 6D). Moreover, the luciferase reporter assays showed that co-transfection with miR-1271-5p mimic and *MTOR* 3’UTR-wt vector significantly reduced the Rluc reporter activity, whereas no reduction in Rluc reporter activity was detected when miR-1271-5p mimic and the mutated vector were co-transfected (Figure 6E, 6F). Additionally, western blot revealed that the protein level of mTOR was negatively correlated with miR-1271-5p expression in PC-3 and DU145 cells (Figure 6G). Taken together, these findings indicate that miR-1271-5p suppresses proliferation, migration, and invasion of PCa cells and that mTOR is a downstream target of miR-1271-5p.

### circMBOAT2 restores mTOR expression by sponging miR-1271-5p

**Figure 7:**
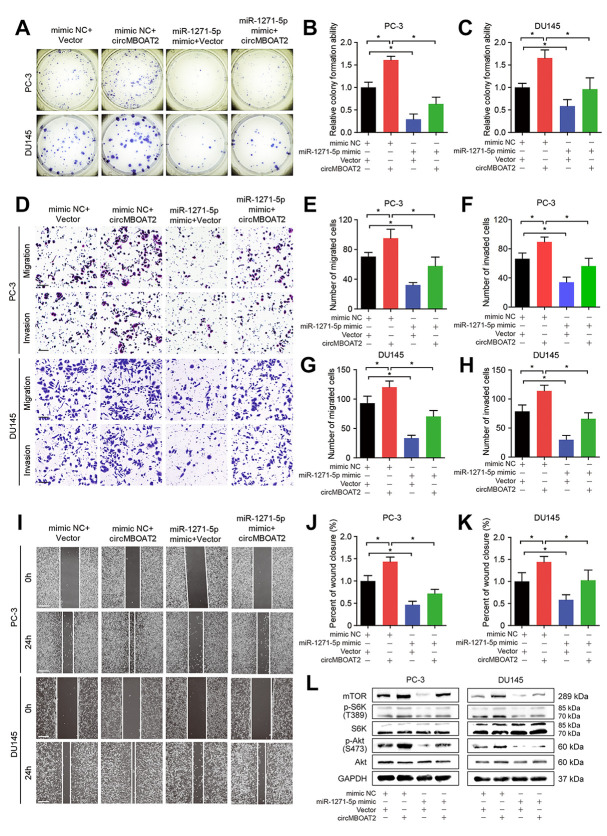
**circMBOAT2 upregulates mTOR expression by sponging miR-1271-5p.** (**A**–**C**) Representative images and quantification of colony formation assays in PC-3 and DU145 cells treated with miR-1271-5p mimic or mimic NC, transfected with vector or circMBOAT2 plasmid. (**D**–**H**) Representative images and quantification of transwell assays in PC-3 and DU145 cells treated with miR-1271-5p mimic or mimic NC, transfected with vector or circMBOAT2 plasmid. Scale bars: 100 μm. (**I**–**K**) Representative images and quantification of wound healing assays in PC-3 and DU145 cells treated with miR-1271-5p mimic or mimic NC, transfected with vector or circMBOAT2 plasmid. Scale bars: 200 μm. (**L**) Western blot analysis of expression of mTOR and its substrates in PC-3 and DU145 cells treated with miR-1271-5p mimic or mimic NC, transfected with vector or circMBOAT2 plasmid. Data are displayed as mean ± SD. *p < 0.05; **p < 0.01.

Next, we further evaluated whether circMBOAT2 can regulate mTOR expression and PCa progression by acting as a sponge for miR-1271-5p. Overexpression of circMBOAT2 overexpression upregulated expression of mTOR, mTORC1 substrate p-S6K, and mTORC2 substrate p-Akt (Figure 7L), indicating activation of the PI3K/Akt pathway, while transfection with miR-1271-5p mimic partly reversed these effects (Figure 7L). Moreover, the CCK-8 and colony formation assays showed that transfection with miR-1271-5p mimic reduced circMBOAT2-induced proliferation in PCa cells (Supplementary Figure 4A, 4B, Figure 7A–7C), suggesting that miR-1271-5p could partly repress the oncogenic role of circMBOAT2. Wound healing assays demonstrated that miR-1271-5p overexpression reversed the enhanced migration induced by circMBOAT2 (Figure 7I–7K). In addition, transwell assays revealed that circMBOAT2 enhanced the migratory and invasive abilities of PCa cells, while miR-1271-5p overexpression attenuated migration and invasion in circMBOAT2-transduced PCa cells (Figure 7D–7H). Altogether, these data confirm that circMBOAT2 upregulates mTOR expression and activates PI3K/Akt signaling to promote proliferation, migration, and invasion of PCa cells by sponging miR-1271-5p.

### Inhibition of mTOR eliminates circMBOAT2-induced tumor progression in PCa

**Figure 8:**
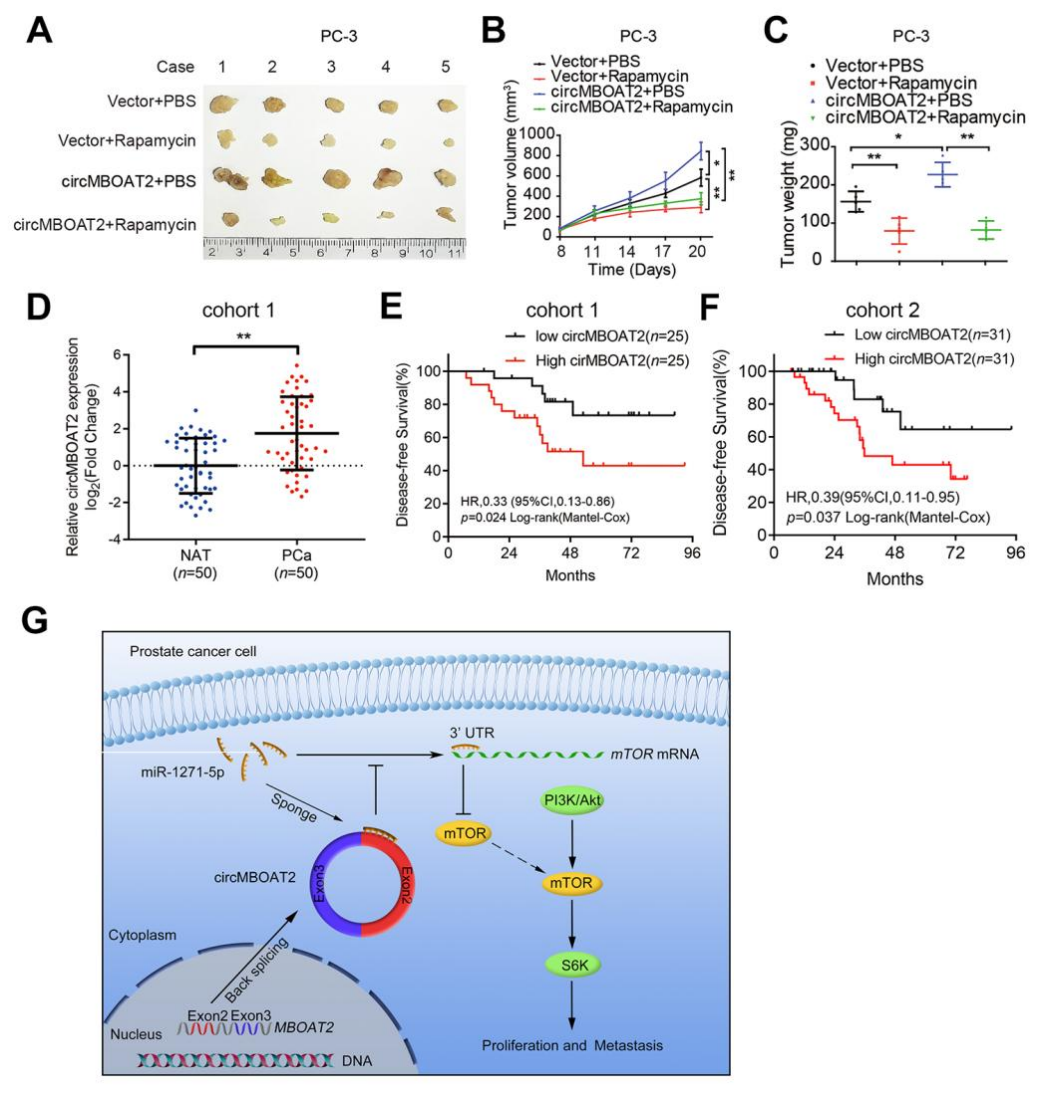
**circMBOAT2 is associated with PCa progression and responds to mTOR inhibitor treatment.** (**A**) Image of the xenograft subcutaneous models of PC-3 cells stably transfected with vector or circMBOAT2 plasmid, treated with rapamycin or PBS. (**B**, **C**) Tumor volumes and weights of subcutaneous xenograft tumors. (**D**) qRT-PCR analysis of circMBOAT2 expression in a cohort of 50 PCa patient tissues paired with their respective NATs. (**E**, **F**) The Kaplan-Meier curves for DFS of PCa patients in cohort 1 and cohort2. Median circMBOAT2 expression levels were used as the cutoff value for patient stratification. (**G**) Proposed model of circMBOAT2-mediated PI3K/Akt activation in PCa tumorigenesis and metastasis. Data are displayed as mean ± SD. *p < 0.05; **p < 0.01.

mTOR is a crucial activator of the PI3K/Akt pathway, and targeting mTOR has been reported as an effective approach to suppress tumor progression. As our results indicated that circMBOAT2 promoted mTOR expression in PCa cells, we investigated whether the oncogenic effects of circMBOAT2 could be eliminated inhibiting mTOR. Western blot showed that the circMBOAT2-induced increase of phosphorylation of S6K and Akt in PC-3 cells were significantly reduced by treatment with rapamycin (Supplementary Figure 5A), a widely used mTOR inhibitor [23]. *In vitro* experiments suggested that rapamycin can reverse the enhanced cell proliferation, migration, and invasion caused by circMBOAT2 (Supplementary Figure 5B–5H). To further evaluate the efficacy of rapamycin on circMBOAT2-induced enhancement of PCa tumor progression *in vivo*, subcutaneous xenograft models were constructed using PC-3 cells stably transfected with vector or circMBOAT2. The tumor-bearing mice were treated with 10 mg/kg rapamycin or PBS. Overexpression of circMBOAT2 promoted tumor growth, and this oncogenic effect of circMBOAT2 was significantly attenuated by rapamycin treatment (Figure 8A–8C). Additionally, Akt inhibitor MK-2206 and PI3K inhibitor GDC-0941 could also rescue the oncogenic effect of circMBOAT2 in PC-3 cells (Supplementary Figure 6A–6J). These results demonstrate that circMBOAT2 promotes PCa progression through an mTOR-dependent pathway, which can be abolished by treatments with PI3K/Akt/mTOR signaling pathway inhibitors.

### circMBOAT2 is overexpressed in PCa and is associated with progression and poor prognosis

circRNAs are recognized as promising biological markers for cancer diagnosis and prognosis [24]. Therefore, we examined whether circMBOAT2 was clinically relevant in PCa. First, we evaluated the expression of circMBOAT2 in a 50-case cohort of PCa tissue and matched normal adjacent tissue (NAT) and found that circMBOAT2 was significantly overexpressed in PCa tissues (Figure 8D). Subsequently, analysis of clinicopathological characteristics of PCa patients in two different cohorts suggested that circMBOAT2 expression was positively associated with high Gleason score and advanced pathological T stage (Tables 1, 2). Moreover, higher circMBOAT2 expression levels were significantly correlated with shorter DFS (Figure 8E, 8F). These findings demonstrate that circMBOAT2 is overexpressed in PCa and that high circMBOAT2 expression correlates with progression and recurrence in PCa.

**Table 1:** Correlation between circMBOAT2 expression and clinicopathologic characteristics of PCa patients in cohort 1.

**Characteristics**	**Patient frequency (%)**	**circMBOAT2 expression level**		
		**Low**	**High**	***p*-value^a^**
**Total cases**	50	25	25	
**Age**				0.382
< 65	19 (38%)	8	11	
≥65	31 (62%)	17	14	
**PSA level (μg/L)**				0.544
≤10	16 (32%)	7	9	
> 10	34 (68%)	18	16	
**Gleason score**				0.012^*^
≤7	36 (72%)	22	14	
> 7	14 (28%)	3	11	
**Pathologic stage**				0.024^*^
T1-2	24 (48%)	16	8	
T3-4	26 (52%)	9	17	
**Lymph-node status**				0.732
Negative	39 (78%)	19	20	
Positive	11 (22%)	6	5	

Abbreviations: ^a^ Chi-square test, ^*^ *p* <0.05, ^**^ *p* <0.01.

**Table 2:** Correlation between circMBOAT2 expression and clinicopathologic characteristics of PCa patients in cohort 2.

**Characteristics**	**Patient frequency (%)**	**circMBOAT2 expression level**		
		**Low**	**High**	***p*-value^a^**
**Total cases**	62	31	31	
**Age**				0.796
< 65	25 (40%)	13	12	
65	37 (60%)	18	19	
**PSA level (μg/L)**				0.409
≤10	19 (31%)	11	8	
> 10	43 (69%)	20	23	
**Gleason score**				0.003^**^
≤7	41 (66%)	26	15	
> 7	21 (34%)	5	16	
**Pathologic stage**				0.022^*^
T1-2	29 (47%)	19	10	
T3-4	33 (53%)	12	21	
**Lymph-node status**				0.520
Negative	50 (81%)	24	26	
Positive	12 (19%)	7	5	

Abbreviations: ^a^ Chi-square test, ^*^ *p* <0.05, ^**^ *p* <0.01.

## DISCUSSION

Patients with advanced PCa frequently have poor prognosis, and current treatments exhibit unsatisfactory efficacy in limiting tumor progression [4, 25]. Therefore, elucidating the molecular mechanisms of PCa tumorigenesis and progression is essential to develop novel therapeutic strategies. Recently, circRNAs have been reported to exert important roles in cancer development [26]. However, the precise mechanisms and functions of circRNAs in PCa have not been explored comprehensively. In the present study, we demonstrated that circMBOAT2 was overexpressed in PCa and correlated with poor prognosis. Loss of function and gain of function experiments revealed that circMBOAT2 promoted proliferation and metastatic behavior of PCa cells *in vitro* and *in vivo*. Mechanistically, circMBOAT2 upregulated mTOR expression and activated PI3K/Akt signaling by acting as a sponge for miR-1271-5p, resulting in increased mTOR expression. These findings provide insight into the oncogenic roles of circRNAs in PCa progression and suggest that inhibition of mTOR may be a potential therapeutic strategy for PCa.

Associations between circRNAs and tumor growth, metastasis, chemoresistance, and poor clinical prognosis have been reported [27–29], suggesting that circRNAs may be exploitable in novel strategies for cancer diagnosis and prognosis. Here, we found that circMBOAT2 was overexpressed in PCa tissues, and higher expression of circMBOAT2 was associated with high Gleason score and advanced pathological T stage. Notably, Kaplan-Meier analysis showed that circMBOAT2 overexpression was associated with significantly shorter DFS in patients with PCa, suggesting that circMBOAT2 may serve as a potential prognostic biomarker for PCa recurrence.

circRNAs function as competing endogenous RNAs (ceRNA) and regulate gene expression by binding to miRNAs, acting as a sponge or decoy. Hu et al. reported that circASAP1 acts as a ceRNA for miR-326 and miR-532-5p, and promoted the progression of liver cancer [30]. Zhang et al. showed that circFGFR1 induced PD-L1 resistance in lung cancer by sponging miR-381-5p [31]. Networks of ceRNAs play crucial roles in the development of multiple cancers, including PCa. In our study, we confirmed the interaction and co-localization between circMBOAT2 and miR-1271-5p, which is a widely known tumor suppressor miRNA [32–39]. Moreover, rescue experiments indicated that circMBOAT2 upregulated mTOR, which in turn promoted PCa cell proliferation, migration, and invasion, while ectopic expression of miR-1271-5p reversed these effects. Our results clarified a ceRNA network that drive the progression of PCa, in which circMBOAT2 regulates mTOR expression by functioning as a ceRNA for miR-1271-5p.

miR-1271-5p has been reported to act as an important tumor suppressor in several tumors [32–39]. Maurel et al. showed that miR-1271-5p was downregulated and suppressed cell proliferation in hepatocellular carcinoma [32]. Zhu et al*.* reported that long non-coding RNA TTN-AS1 promoted cell proliferation and migration by inhibiting miR-1271-5p in PCa cells [36]. However, the clinical significance and biological function of miR-1271-5p in PCa remain largely unknown. In the present study, we found that miR-1271-5p suppressed the proliferation, migration and invasion of PCa cells by targeting mTOR. Moreover, miR-1271-5p was significantly downregulated in PCa tissues and low miR-1271-5p expression correlated with recurrence in PCa.

Another important finding in our study was that circMBOAT2 could activate the PI3K/Akt pathway. The PI3K/Akt pathway is often aberrantly activated in PCa, especially in metastatic PCa [16]. Hyperactivation of PI3K/Akt signaling promotes the proliferation, metastasis, and recurrence of PCa [13]. Although several studies have uncovered sophisticated regulatory networks involving the PI3K/Akt pathway, the roles of circRNAs in PI3K/Akt activation in PCa remains unknown. In our study, we found that circMBOAT2 upregulated the expression of mTOR, a significant component in the PI3K/Akt pathway, resulting in subsequent activation of the PI3K/Akt pathway by phosphorylating the downstream effectors Akt and S6K. Furthermore, blocking the PI3K/Akt pathway with rapamycin, an mTOR inhibitor, abolished circMBOAT2-induced PCa progression. These results elucidate the mechanisms by which circRNA serve as essential regulators of PI3K/Akt pathway, and support the role of circMBOAT2 as a potential target for intervention in PCa.

Since the PI3K/Akt pathway plays a key role in the development of many cancers, including PCa, inhibition of mTOR is widely considered a promising treatment many cancer patients [40–42]. A phase II clinical trial demonstrated that treatment with the mTOR inhibitor voxtalisib achieved satisfactory efficacy in patients with follicular lymphoma [43]. Mohlin et al. showed that treatment with the mTOR inhibitor IBL-302 inhibited the growth of neuroblastoma in animal models [44]. Although several mTOR inhibitors are available for cancer therapy, clinical trials using mTOR inhibitors have showed disappointing responses in PCa [45–46]. The inadequate knowledge of effective indicators for treatment with mTOR inhibitors has limited their utility in PCa patients. In the present study, we demonstrated that circMBOAT2 sponged miR-1271-5p and upregulated mTOR expression, which ultimately induced the progression of PCa. Inhibition of mTOR with rapamycin suppressed circMBOAT2-induced proliferation, migration, and invasion of PCa cells *in vitro*. Importantly, administration of rapamycin significantly reduced tumor burden in circMBOAT2-transduced PCa xenograft murine tumor models. Therefore, elucidating the role of circMBOAT2 in PCa demonstrates that circMBOAT2 may be a potential indicator for mTOR-targeting as an intervention in PCa.

In summary, our findings revealed that circMBOAT2 was overexpressed in PCa tissues and high expression of circMBOAT2 was associated with poor prognosis in patients with PCa. We also demonstrated that circMBOAT2 promoted proliferation and metastasis of PCa through miR-1271-5p/mTOR axis-mediated activation of the PI3K/Akt pathway. Our study not only provides an insight into the oncogenic role of circRNAs in the development and progression of PCa, but also highlights circMBOAT2 as a potential prognostic biomarker and promising therapeutic target in PCa.

## MATERIALS AND METHODS

### Patients and clinical samples

A total of 50 pairs of tumor tissue and normal adjacent tissue (NAT) samples (termed cohort 1) as well as 62 cases of tumor tissue sample (termed cohort 2) were collected from patients with PCa who underwent radical resection surgery at Sun Yat-sen Memorial Hospital, Sun Yat-sen University (Guangzhou, China). All samples were defined by pathological diagnosis. The study was approved by the Ethical Review Committee of Sun Yat-sen Memorial Hospital. Informed consent was obtained from each patient prior to sample collection.

### Cell lines and cell culture

The human PCa cell lines PC-3, DU145, VCaP, LNCaP, and C4-2B, and the human normal prostate epithelial cell line RWPE-1, were purchased from American Type Culture Collection (ATCC, VA, USA). PC-3 cells were cultured in F12-K medium (Gibco, NY, USA). DU145 and VCaP cells were cultured in DMEM (Gibco, NY, USA). LNCaP and C4-2B cells were cultured in RPMI 1640 medium (Gibco, NY, USA). RWPE-1 cells were cultured in Keratinocyte Serum Free Medium (Gibco, NY, USA). All media were supplemented with 100 U/ml penicillin, 100 μg/ml streptomycin and 10% fetal bovine serum (FBS). All cells were cultured in an incubator with 5% CO_2_ at 37°C.

### cDNA synthesis and quantitative real-time PCR (qRT-PCR)

For gene expression analysis, cDNA was synthesized with the PrimeScript RT Reagent Kit (Takara, Shiga, Japan). The cDNA samples were mixed with TB Green Premix Ex Taq II (Takara, Shiga, Japan) to perform qRT-PCR. The relative expression levels were calculated by the 2 ^-ΔΔ^CT method. *GAPDH* was used as an internal control for circRNA and mRNA expression, and *U6* was used as an internal control for microRNA expression. All primers are listed in Supplementary materials; Supplementary Table 1.

### Actinomycin D assay

PC-3 and DU145 cells were treated with 2 μg/ml actinomycin D (Sigma -Aldrich, MO, USA). The cells were harvested at the indicated time points and total RNA was extracted. The stability of circMBOAT2 and *MBOAT2* mRNA was analyzed by qRT-PCR.

### RNase R treatment

For RNase R treatment, 2 μg of total RNA per sample was treated for 30 min at 37°C with RNase R (3 U/μg) (Epicentre Technologies, WI, USA), or buffer only as control, then purified with a RNeasy MinElute Cleanup Kit (QIAGEN, Hilden, Germany). Finally, the samples were subjected to reverse transcription and subsequently analyzed with qRT-PCR, as described above.

### Animal experiments

For the PC-3 cell line xenograft subcutaneous model, 3 × 10^6^ stable luciferase-labeled PC-3 cells with sh-MBOAT2 or sh-NC were subcutaneously injected into BALB/c nude mice (Sun Yat-sen University, Guangzhou, China). The volumes of tumors were recorded beginning 8 days after injection. After 23 days the mice were euthanized. Tumor samples were fixed in 37% formalin and embedded in paraffin for subsequent histological analysis.

For the PC-3 cell line xenograft metastatic model, 1 × 10^6^ stable luciferase-labeled PC-3 cells with the circMBOAT2 expression vector or empty vector were injected into the tail vein of BALB/c nude mice. After 4 weeks the lungs were excised and analyzed by fluorescence and a small animal imaging system (Neopanora, Zhuhai, China).

For rapamycin treatment, xenograft subcutaneous models were constructed with stable luciferase-labeled PC-3 cells with circMBOAT2 or Vector. Beginning seven days after injection, the mice were intratumorally injected with PBS or rapamycin (10 mg/kg) every 3 days, respectively. Two weeks after treatment initiation, the tumors were excised and tumor weight and volume were recorded.

All animal experiments were conducted with the approval of the Animal Ethics Committee of Sun Yat-sen University.

### HE and immunohistochemistry

For HE, sections were stained with hematoxylin and eosin. For IHC, rehydrated sections were staining according to published methods [47]. Images were captured with a Leica DM2000 microscope. The following antibodies and dilutions were used: anti-Ki67 antibody (ab15580, 1:2000) and goat anti-rabbit IgG H&L (HRP) (ab6721, 1:500) were purchased from Abcam (Cambridge, UK).

### Biotin-labeled RNA pull-down

The biotin-labeled probes for circMBOAT or NC were synthesized (GenePharma Shanghai, China) and incubated with streptavidin magnetic beads (Thermo Fisher Scientific, MA, USA) at 25°C for 2 h to form probe-coated beads. The probe-coated beads were incubated with PCa cell lysates at 4°C overnight and then separated for RNA extraction, cDNA synthesis, and qRT-PCR. All probes are detailed in Supplementary materials; Supplementary Table 2.

### Biotin-labeled RNA capture

PCa cells were transfected with biotinylated biotin-labeled miR-1271-5p or miR-NC and harvested after 48 h. The cells were lysed and incubated with streptavidin magnetic beads followed by separation and analysis, as mentioned above. All miRNA mimics are described in Supplementary materials; Supplementary Table 2.

### Western blot

Western blot was performed as previously described [48]. The following antibodies and dilutions were used: mTOR antibody (2983, 1:1000), phospho-mTOR (Ser2448) antibody (5536, 1:1000), Akt (pan) antibody (4691, 1:1000), phospho-Akt (Ser473) antibody (4060, 1:1000), p70 S6 kinase antibody (2708, 1:1000), phospho-p70 S6 kinase (Thr389) antibody (9234, 1:1000), and anti-rabbit IgG HRP-linked antibody (7074, 1:5000) were purchased from Cell Signaling Technology (MA, USA). Anti-GAPDH antibody (ab181602, 1:10,000) was purchased from Abcam (Cambridge, UK).

### Bioinformatics analysis

Potential target miRNAs of circMBOAT2 were obtained from the MiRanda, TargetScan, and RNAhybrid databases. The miR-1271-5p binding sites in circMBOAT2 were predicted using the RNAalifold webtool. The binding sites for miR-1271-5p and the 3’UTR of *MTOR* were predicted by starBase v2.0 [49] and RegRNA tools.

### Statistics

Statistical analyses were evaluated using SPSS v.20.0 (IBM, IL, USA). All experiments were performed in triplicate and the results are presented as the means ± SD. Two-tailed Student’s *t* test, Mann-Whitney *U* test, and one-way ANOVA were used to determine statistical significance, as appropriate. The correlations were measured by Pearson's correlation test. The *χ*^2^ test was applied to analyze the correlation between circMBOAT2 levels and clinicopathological characteristics of patients with PCa. Disease-free survival (DFS) was evaluated by the Kaplan-Meier method and analyzed using the log-rank test. Univariate and multivariate analysis were performed to identify risk factors for DFS of PCa patients. A P-value of *p* < 0.05 was considered statistically significant.

## Supplementary Material

Supplementary Methods

Supplementary Figures

Supplementary Tables
