# Supplementary material for: Circular RNA circMBOAT2 promotes prostate cancer progression via a miR-1271-5p/mTOR axis
Source: Aging (Albany NY). 2020 Jul 9;12(13):13255–80. doi: 10.18632/aging.103432 (PMC7377862; doi:10.18632/aging.103432)
Supplement: Supplementary Methods [file aging-12-103432-s001..pdf]

SUPPLEMENTARY METHODS

Nucleic acids extraction

Total RNA was isolated with the RNAiso Plus (TaKaRa, Japan) and Genomic DNA (gDNA) was extracted with TaKaRa MiniBEST Universal Genomic DNA Extraction Kit (Takara, Japan) following the manufacturer's instructions. PARIS Kit (Thermo Fisher Scientific, MA, USA) as used for separation of cytoplasmic and nuclear fractions prior to RNA extraction.

Fluorescence in situ hybridization

PC-3 cells were seeded in confocal dishes and then hybridized with Cy3-labeled circMBOAT2 probe, Cy3-labeled *U6* snRNA probe, Cy3-labeled *18s* rRNA probe, FAM3-labeled-miR-1271-5p probe using Fluorescent in Situ Hybridization Kit (GenePharma, Shanghai, China). All probes were synthesized by GenePharma (Shanghai, China) and showed in Supplementary Table 2. Nuclei were counterstained with DAPI. The Specimens were analyzed with ZEISS LSM800 confocal microscope (Carl Zeiss AG, Oberkochen, Germany).

RNA interference, plasmid construction and transfection

Small interfering RNAs (siRNAs) for circMBOAT2 were designed and synthesized by GenePharma (Shanghai, China). miRNA mimics and inhibitors were obtained from RiboBio (Guangzhou, China). Transfection was performed using Lipofectamine RNAiMAX (Thermo Fisher Scientific, MA, USA) following manufacturer's protocol. The full-length circMBOAT2 was cloned into the plenti-ciR-mCherry-T2A™ vector (IGE BIO, Guangzhou, China). The pLKO.1-Puro vector was used to generate a short hairpin RNA (shRNA) against circMBOAT2. The pCDH-CMV-MCS-EF1-GFP for luciferase overexpression was constructed. Lentivirus was packaged in HEK 293T cells and infected PC-3 and DU145 cells, which were subsequently selected with flow cytometry and 1 μg/ml puromycin for 1 week. Oligos were showed in Supplementary Table 3.

CCK-8, colony formation and EdU assays

For CCK-8 assays, 2×10^3 PCa cells were seeded in 96-well plates and incubated with CCK-8 solution (APExBIO, TX, USA) every 24 h for 5 days according to the manufacturer's instructions. For colony formation assays, 1×10^3 PCa cells were seeded and cultured in 6-well plates for 2 weeks. The colonies were fixed in 4% paraformaldehyde solution and stained with 0.1% crystal violet. For EdU assays, PCa cells were seeded into 96-well plates and cultured for 24 h. Cells were labeled with EdU kit (RioboBio, Guangzhou, China) according to the manufacturer's instructions. The images were captured with ImageXpress Mirco Confocal (Molecular Devices, CA, USA).

Wound healing and transwell assays

For wound healing assays, PCa cells were seeded into 6-well plates and scratched lines were made using 200 μL pipette tips on cell layers. Images of wounds were obtained at indicated time with an Olympus IX2 inverted microscope (Olympus, Tokyo, Japan). For Transwell migration and invasion assays, 5×10^4 PCa cells suspended in serum-free medium were seeded into the 24-well upper chambers of Transwell apparatus (Corning, MA, USA) with or without pre-coated Matrigel (Corning, MA, USA). Medium containing 10% FBS was added to the lower chambers. After 24 h (for DU145 cells) or 48 h(for PC-3 cells) incubation, the cells were fixed in 4% paraformaldehyde solution and stained with 0.1% crystal violet. The number of cells migrated to the lower chamber were counted in five random fields under a Leica DM2000 microscope (Leica Camera AG, Wetzlar, Germany).

Dual-luciferase reports assay

Full length of circMBOAT2 and the 3' UTR fragments of *MTOR* were cloned into the psi-CHECK2 plasmid. Plasmids with mutant binding site of miR-1271-5p were used as a negative control. All plasmids and miR-1271-5p were transfected into HEK 293T cells and the luciferase activities were measured using a Dual-Luciferase Reporter Assay System (Promega, WI, USA) referencing to the manufacturer's protocol 48 h after transfection. Renilla luciferase (Rluc) intensity was normalized with firefly luciferase intensity.
