# Supplementary material for: Circular RNA circMBOAT2 promotes prostate cancer progression via a miR-1271-5p/mTOR axis
Source: Aging (Albany NY). 2020 Jul 9;12(13):13255–80. doi: 10.18632/aging.103432 (PMC7377862; doi:10.18632/aging.103432)
Supplement: Supplementary Figures [file aging-12-103432-s003..pdf]

SUPPLEMENTARY FIGURES

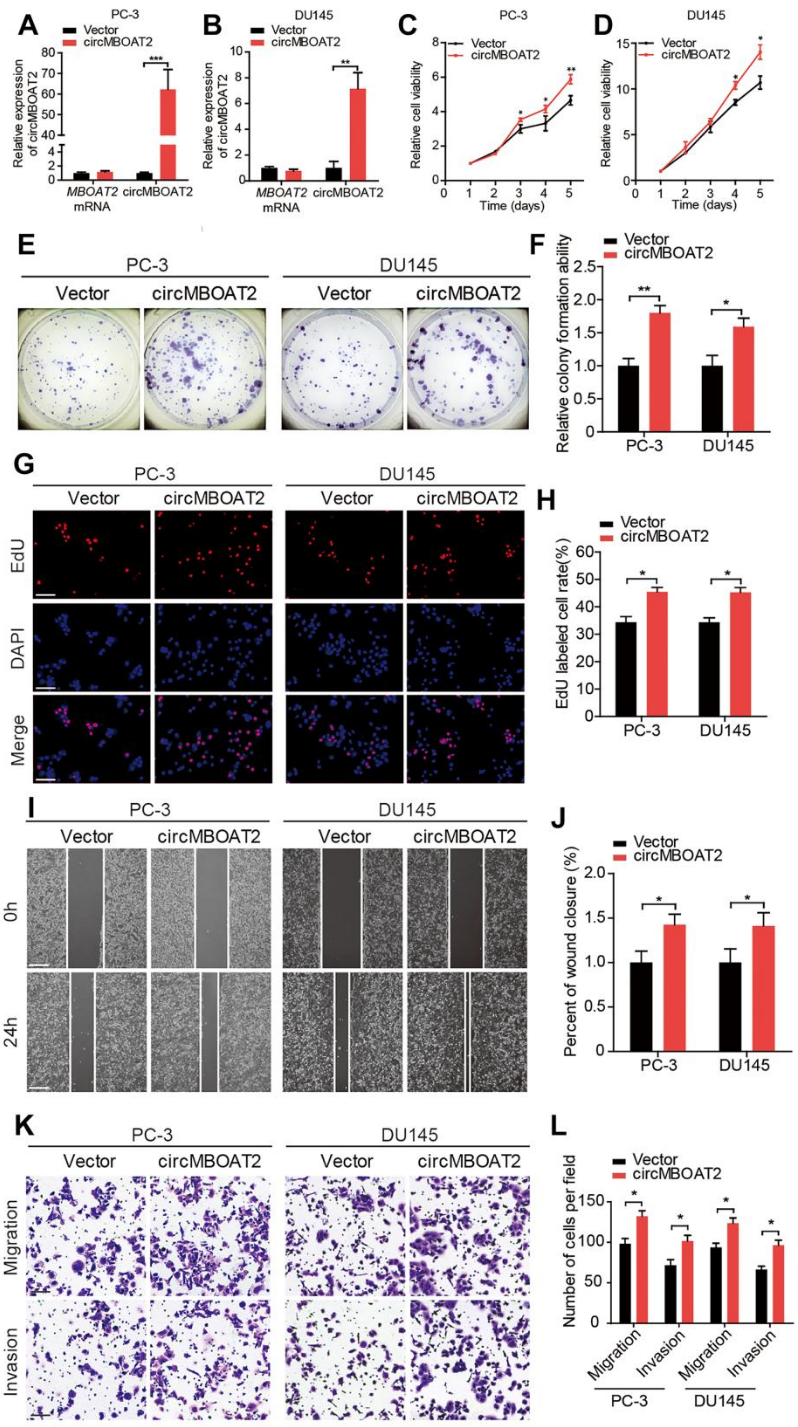

Supplementary Figure 1. circMBOAT2 exerts oncogenic effects in PCa cells. (**A**, **B**) qRT-PCR analysis of circMBOAT2 and MBOAT2 in PC-3 and DU145 cells treated with circMBOAT2 plasmid. (**C**, **D**) CCK-8 assay determined the cell viability in PC-3 and DU145 cells treated with circMBOAT2 plasmid. (**E**, **F**) Representative images and quantifications of colony formation assays in PC-3 and DU145 cells treated with circMBOAT2 plasmid. (**G**, **H**) Representative images and quantifications of EdU assays in PC-3 and DU145 cells treated with circMBOAT2 plasmid. Scale bars: 100 μm. (**I**, **J**) Representative images and quantifications of wound healing assays in PC-3 and DU145 cells treated with circMBOAT2 plasmid. Scale bars: 200 μm. (**K**, **L**) Representative images and quantifications of transwell assays in PC-3 and DU145 cells treated with circMBOAT2 plasmid. Scale bars: 100 μm. Data are displayed as mean ± SD. *$p < 0.05$; **$p < 0.01$; ***$p < 0.01$.

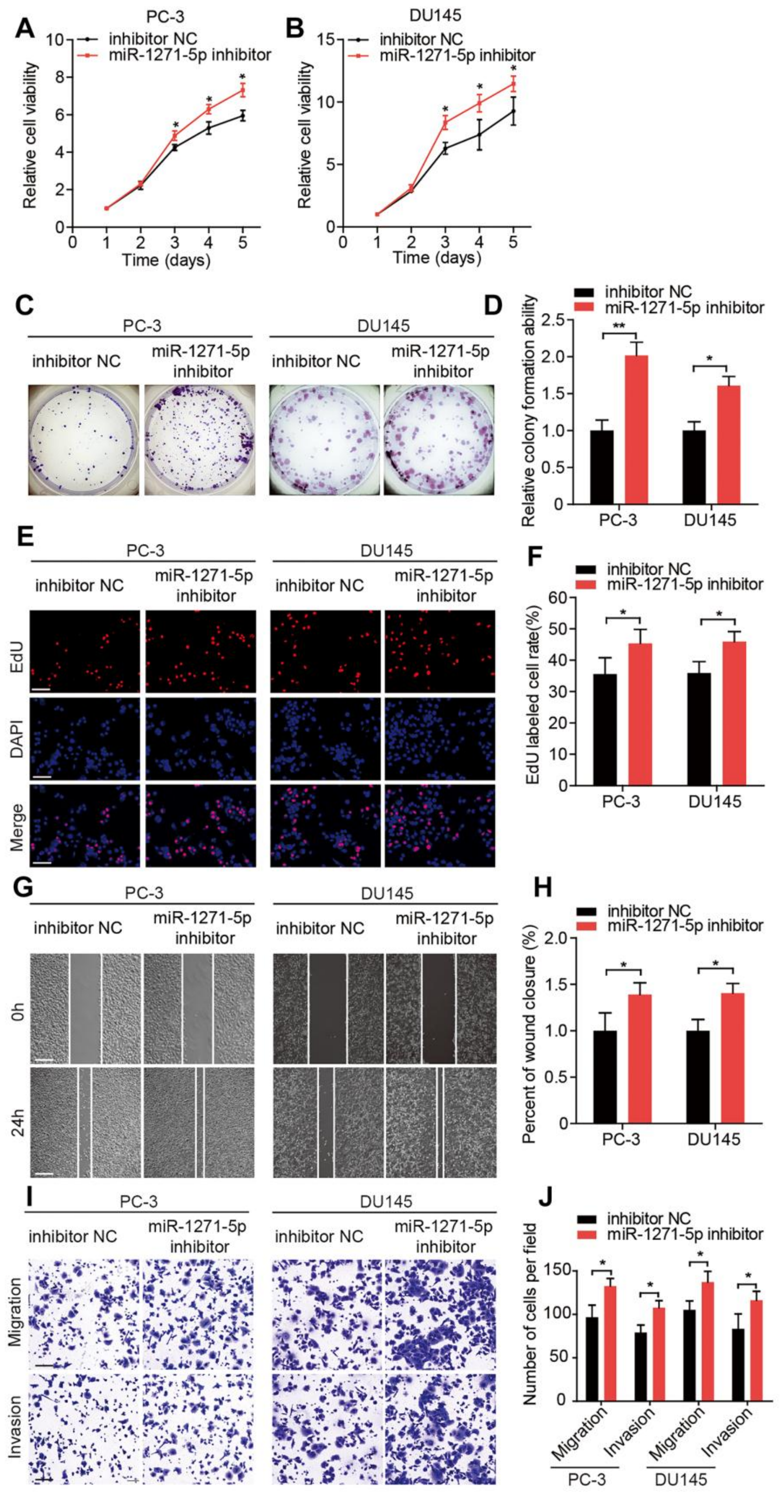

Supplementary Figure 2. Silencing miR-1271-5p promotes cell proliferation, migration and invasion in PCa cells. (**A**, **B**) CCK-8 assay determined the cell viability in PC-3 and DU145 cells treated with miR-1271-5p inhibitor. (**C**, **D**) Representative images and quantifications of colony formation assays in PC-3 and DU145 cells treated with miR-1271-5p inhibitor. (**E**, **F**) Representative images and quantifications of EdU assays in PC-3 and DU145 cells treated with miR-1271-5p inhibitor. Scale bars: 100 μm. (**G**, **H**) Representative images and quantifications of wound healing assay in PC-3 and DU145 cells treated with miR-1271-5p inhibitor. Scale bars: 200 μm. (**I**, **J**) Representative images and quantifications of transwell assay in PC-3 and DU145 cells treated with 1271-5p inhibitor. Scale bars: 100 μm. Data are displayed as mean ± SD. *p < 0.05; **p < 0.01.

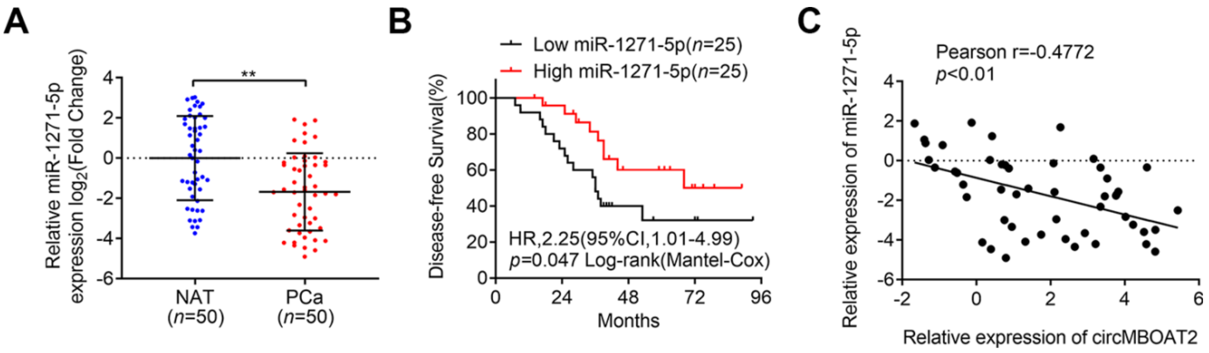

Supplementary Figure 3. The abundance and clinical significance of miR-1271-5p in PCa patients. (**A**) qRT-PCR analysis of miR-1271-5p expression in cohort 1 of 50 PCa patient tissues paired with their respective NATs. (**B**) The Kaplan-Meier curves for DFS of PCa patients in cohort 1. Median miR-1271-5p expression levels were used as the cutoff value for patient stratification. (**C**) The correlation between miR-1271-5p and circMBOAT2 in PCa tissues (*n*=50). Data are displayed as mean ± SD. $^{**}p < 0.01$.

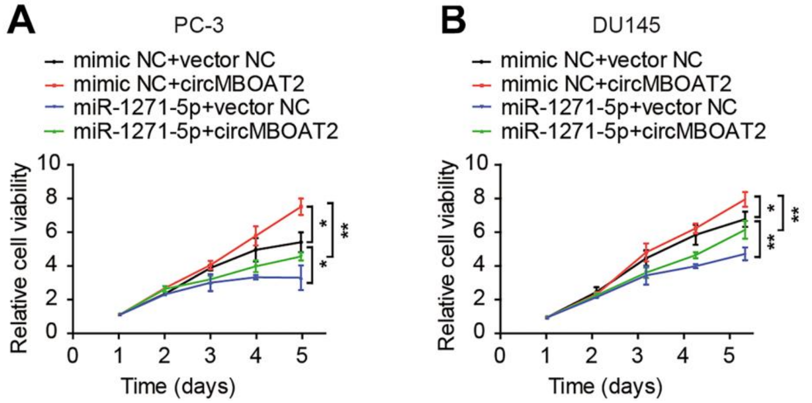

Supplementary Figure 4. circMBOAT2 promotes cell proliferation by sponging miR-1271-5p in PCa cells. (**A**, **B**) CCK-8 assay determined the cell viability in PC-3 and DU145 cells treated with miR-1271-5p mimic or mimic NC, transfected with vector or circMBOAT2 plasmid. Data are displayed as mean ± SD. $^{*}p < 0.05$; $^{**}p < 0.01$.

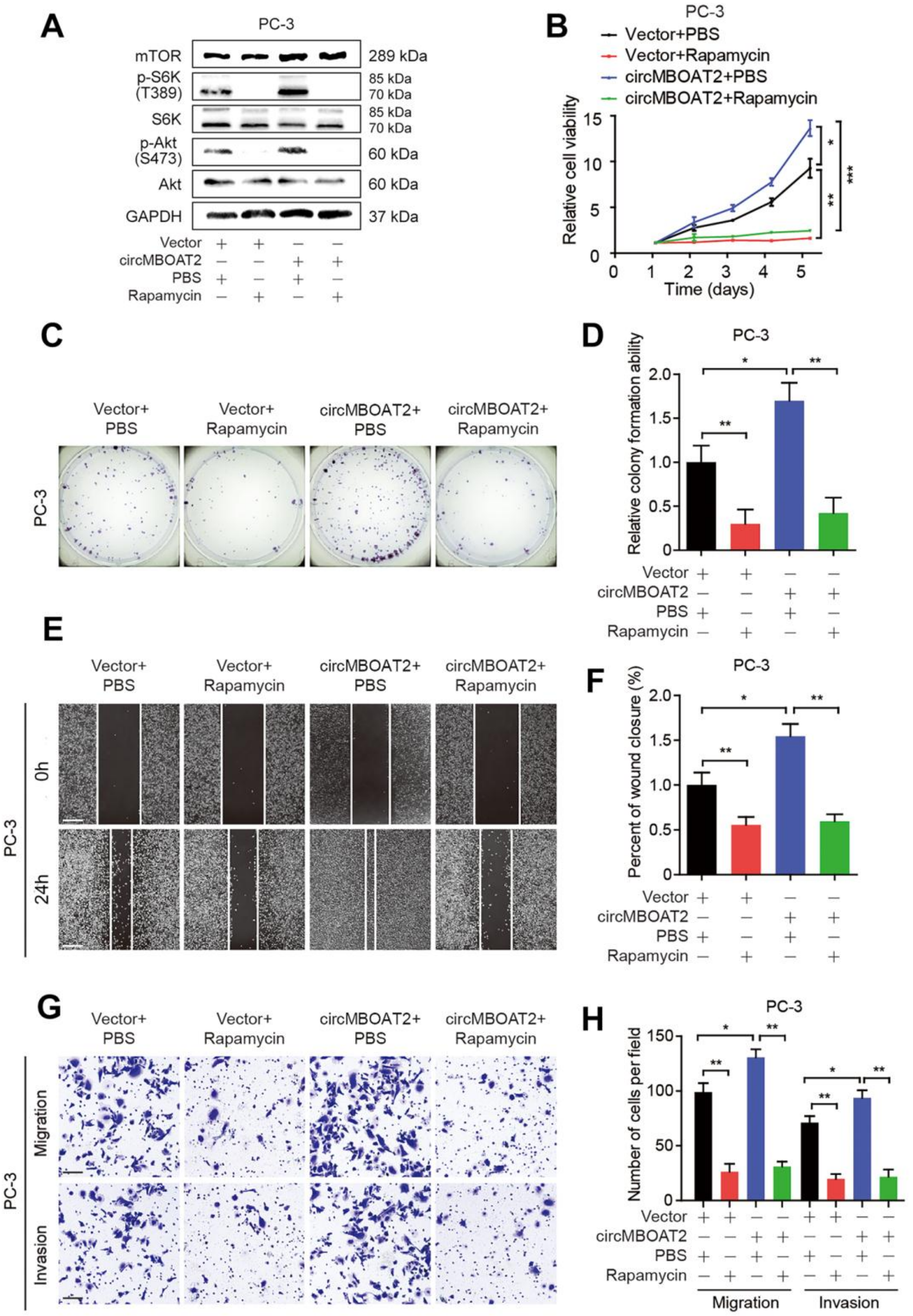

Supplementary Figure 5. Rapamycin abolishes the oncogenic role of circMBOAT2 in PCa cells. (**A**) Western blot analysis of expression of mTOR and its substrates in PC-3 cells transfected with vector or circMBOAT2 plasmid, treated with 100 nM rapamycin or PBS for 48h. (**B**) CCK-8 assay determined the cell viability in PC-3 cells transfected with vector or circMBOAT2 plasmid, treated with 100 nM rapamycin or PBS. (**C**, **D**) Representative images and quantifications of colony formation assays in PC-3 cells transfected with vector or circMBOAT2 plasmid, treated with 100 nM rapamycin or PBS. (**E**, **F**) Representative images and quantifications of wound healing assays in PC-3 cells transfected with vector or circMBOAT2 plasmid, treated with 100 nM rapamycin or PBS. Scale bars: 200 μm. (**G**, **H**) Representative images and quantifications of transwell assay in PC-3 cells transfected with vector or circMBOAT2 plasmid, treated with 100 nM rapamycin or PBS. Scale bars: 100 μm. Data are displayed as mean ± SD. *$p < 0.05$; **$p < 0.01$; ***$p < 0.01$.

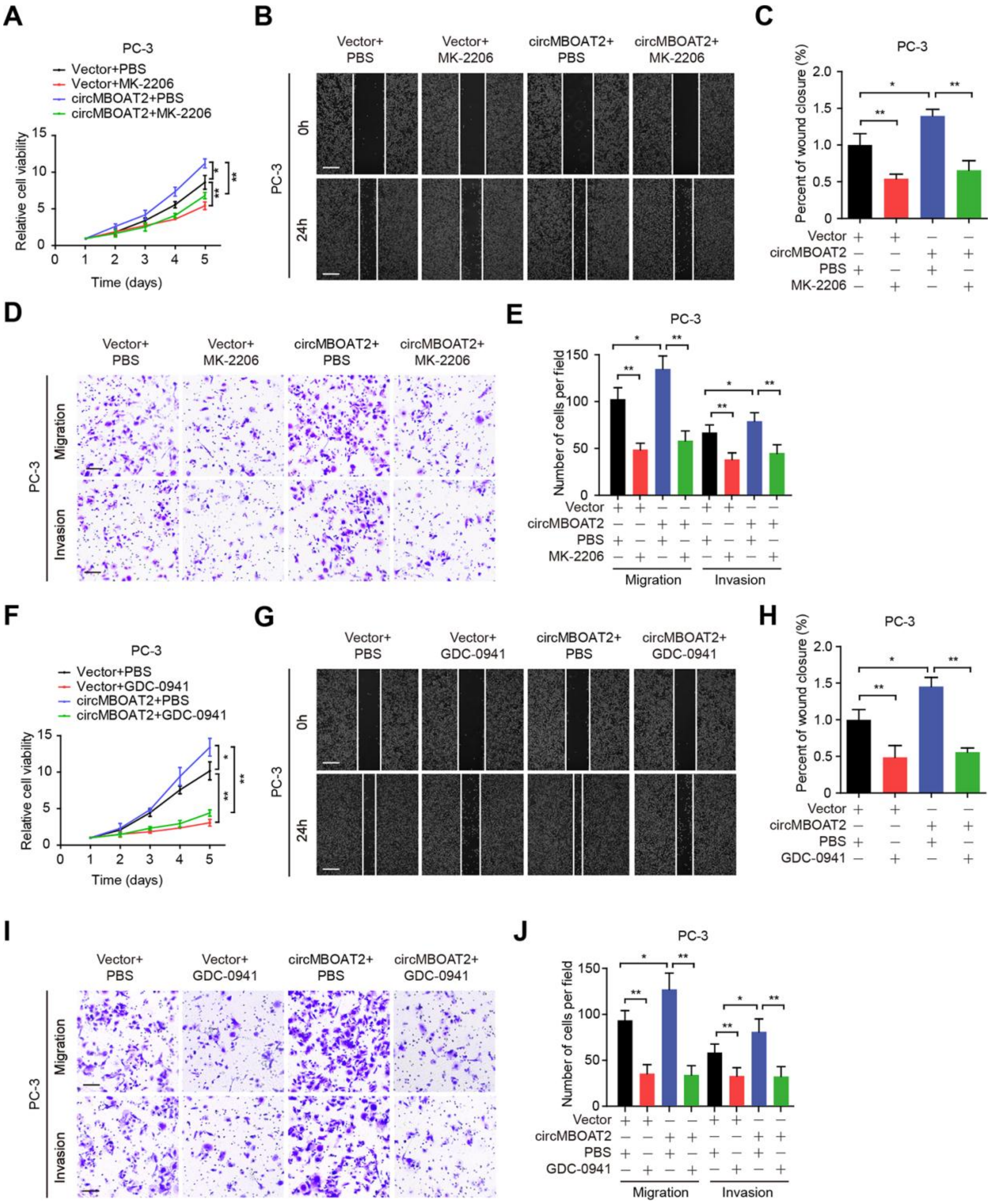

Supplementary Figure 6. MK-2206 and GDC-0941 abolish the oncogenic role of circMBOAT2 in PCa cells. (**A**) CCK-8 assay determined the cell viability in PC-3 cells transfected with vector or circMBOAT2 plasmid, treated with 1 uM MK-2206 or PBS. (**B**, **C**) Representative images and quantifications of wound healing assay in PC-3 cells transfected with vector or circMBOAT2 plasmid, treated with 1 μM MK-2206 or PBS. Scale bars: 200 μm. (**D**, **E**) Representative images and quantifications of transwell assay in PC-3 cells transfected with vector or circMBOAT2 plasmid, treated with 1 μM MK-2206 or PBS. Scale bars: 200 μm. (**F**) CCK-8 assay determined the cell viability in PC-3 cells transfected with vector or circMBOAT2 plasmid, treated with 1 μM GDC-0941 or PBS. (**G**, **H**) Representative images and quantifications of wound healing assay in PC-3 cells transfected with vector or circMBOAT2 plasmid, treated with 1 μM GDC-0941 or PBS. Scale bars: 200 μm. (**I**, **J**) Representative images and quantifications of transwell assay in PC-3 cells transfected with vector or circMBOAT2 plasmid, treated with 1 μM GDC-0941 or PBS. Scale bars: 200 μm. Data are displayed as mean ± SD. *$p < 0.05$; **$p < 0.01$.
