# Supplementary material for: Circular RNA circMBOAT2 promotes prostate cancer progression via a miR-1271-5p/mTOR axis
Source: Aging (Albany NY). 2020 Jul 9;12(13):13255–80. doi: 10.18632/aging.103432 (PMC7377862; doi:10.18632/aging.103432)
Supplement: Supplementary Tables [file aging-12-103432-s002..pdf]

SUPPLEMENTARY TABLES

Supplementary Table 1. The primers used in this study are listed as follows.

Primers	Sequence (5'-3')
circMBOAT2 Forward	TGGGCCTTTATCTTGCACTT
circMBOAT2 Reverse	AGGCAAAGAGTTGGCACACTA
MBOAT2 Forward	GGGTATGACGAAAATGGAGCAGC
MBOAT2 Reverse	CTTTTGAGCCAAAGAGCTGTCTG
GAPDH Forward	CAAGGCTGAGAACGGGAAG
GAPDH Reverse	TGAAGACGCCAGTGGACTC
MTOR Forward	AGCATCGGATGCTTAGGAGTGG
MTOR Reverse	CAGCCAGTCATCTTTGGAGACC
CAMK2N1 Forward	GGACACCAACAACTTCTTCGGC
CAMK2N1 Reverse	GTCGGTCATATTTTTCAGCACGTC
BSDC1 Forward	CTGAGCCCTATGATGGCACCAA
BSDC1 Reverse	ACTGGGAAAGCCAGGCGTCAAA
ZCCHC11 Forward	TGCTCAACAGGTGGCTGGTTCA
ZCCHC11 Reverse	GAGTTCTGTGGAAATGGCTGAGG
miR-1271-5p Forward	ACACTCCAGCTGGGCTTGGCACCTAGCAAG
miR-330-3p Forward	ACACTCCAGCTGGGGCAAAGCACACGGCCTG
miR-454 Forward	ACACTCCAGCTGGGTAGTGCAATATTGCTTA
miR-3666 Forward	ACACTCCAGCTGGGCAGTGCAAGTGTAGA
miR-889 Forward	ACACTCCAGCTGGGAATGGCTGTCCGTAGT
miRNA Reverse	GTGCAGGGTCCGAGGT
U6 Forward	CGCTTCGGCAGCACATATAC
U6 Reverse	TTCACGAATTTGCGTGTCAT
18S rRNA Forward	GGAGTATGGTTGCAAAGCTGA
18S rRNA Reverse	TCCTGCTTTGGGGTTCGATT

Supplementary Table 2. The probes used in this study are listed as follows.

FISH Probes	Sequence (5'-3')
Cy3-*U6*	TTTGCGTGTCATCCTTGCG
Cy3-*18S*	CTTCCTTGGATGTGGTAGCCGTTTC
Cy3-circMBOAT2	UGGCACACUACAAAGUUGACUUGUGCAUGUUCUCCACUCC
FAM 3-miR-1271-5p	TGAGTCTTGCTAGGTGCCAAG
Biotin-labeled probes	
Biotin-NC	CUGUACUUGUUCCAACUCAAGUGCUAUACUUGGUAGAUCAGA
Biotin-circMBOAT2	UGGCACACUACAAAGUUGACUUGUGCAUGUUCUCCACUCC
Biotin-miR-1271-5p mimic	CUUGGCACCUAGCAAGCACUCA
Biotin-mimic NC	CAGUACUUUUGUGUAGUACAA

Supplementary Table 3. The oligonucleotides transfected in this study are listed as follows.

Oligonucleotides	Sequence (5'-3')
si-NC sense	UUCUCCGAACGUGUCACGUTT
si-NC antisense	ACGUGACACGUUCGGAGAATT
si-circMBOAT2#1 sense	CAUGCACAAGUCAACUUUGTT
si-circMBOAT2#1 antisense	CAAAGUUGACUUGUGCAUGTT
si-circMBOAT2#2 sense	CACAAGUCAACUUUGUAGUTT
si-circMBOAT2#2 antisense	ACUACAAAGUUGACUUGUGTT
sh-circMBOAT2#1 sense	CAUGCACAAGUCAACUUUGTT
sh-circMBOAT2#1 antisense	CAAAGUUGACUUGUGCAUGTT
mimic NC sense	UUCUCCGAACGUGUCACGUTT
mimic NC antisense	ACGUGACACGUUCGGAGAATT
miR-1271-5p mimic sense	CUUGGCACCUAGCAAGCACUCA
miR-1271-5p mimic antisense	AGUGCUUGCUAGGUGCCAAGUU
inhibitor NC	CAGUACUUUUGUGUAGUACAA
miR-1271-5p inhibitor	ACGUGACACGUUCGGAGAATT
